# Value-Sensitive Design of Potable Water Reuse: Aligning Academic Research with Societal Concerns

**DOI:** 10.1007/s11948-025-00542-3

**Published:** 2025-07-01

**Authors:** Karen Moesker, Martijn Wiarda

**Affiliations:** 1 Department of Values, Technology and Innovation, Delft University of Technology, Jaffalaan 5, Delft, 2628 BX The Netherlands; 2 Department of Industrial Engineering and Innovation Sciences, Eindhoven University of Technology, Group Technology, Innovation & Society, Het Eeuwsel 53, Eindhoven, 3600 MB The Netherlands

**Keywords:** Responsible research and innovation, Value-sensitive design, Water recycling, Direct potable reuse, Indirect potable reuse, Advanced water purification

## Abstract

As global water scarcity worsens, potable water reuse is increasingly considered a vital solution for augmenting water supplies. However, public acceptance remains a significant barrier, presumably because of a misalignment between the public values reflected by these systems and those that are held by the communities that these systems intend to serve. This study explores this potential misalignment by systematically identifying and analysing the most prevalent values inscribed in academic research on potable water reuse. We employ a mixed-methods approach, combining probabilistic topic modelling with thematic analysis of 2940 academic publications to identify and conceptualise latent values discussed in the literature. Our findings suggest that the values ‘reliability’, ‘sustainability’, ‘health’, and ‘safety’ are most prevalent but that their conceptualisation remains largely ambivalent. For example, sustainability exhibits an ambivalent relationship with safety, sometimes conflicting and sometimes supporting, depending on the research perspective. Crucially, this research demonstrates a predominantly technocentric understanding of these values. While this technical focus is undeniably important, it also risks overlooking broader societal concerns and other value interpretations. This research highlights the need for a more value-sensitive approach to ensure a more responsible potable water reuse, incorporating a wider range of public values to promote the system’s social and ethical desirability.

## Introduction

As global water scarcity intensifies and demand continues to rise, there has been a notable surge in the exploration of sustainable water management practices in recent decades. Among the potential strategies in this pursuit, water reuse is a potential solution for conserving and efficiently utilising this finite resource. However, water reuse systems, particularly those for potable applications (henceforth potable water reuse), persistently encounter resistance (Lee & Jepson, 2020) despite the continuous development of approaches fostering acceptance (e.g. Furlong et al., 2019; Katz & Tennyson, 2015; Wilcox et al., 2016).

One reason for this may be that many approaches rely on the disputed information deficit model, assuming the main underlying issue of public opposition is a lack of information about the technology (Moesker et al., 2024). This reductionistic perspective is at odds with insights from Value-Sensitive Design (VSD) – and Responsible Research and Innovation (RRI) in a broader sense – suggesting that public opposition arises when the technological design fails to accommodate relevant values (Taebi et al., 2014). Values can be regarded as critical aspects of life (Friedman et al., 2013), shaped by societal beliefs of what is worth striving for, and essentially serve as “orienting judgment devices” for present and future actions (van de Poel & Kudina, 2022). However, addressing all pertinent values can be demanding, partly because values can be conceptualised and can conflict in various ways, requiring design adaptations or value trade-offs (Taebi et al., 2014).

The importance of values in the water management sector is increasingly acknowledged, highlighted by the recently published United Nations World Water Development Report titled ‘Valuing Water’ (2024) and the rising interest in water ethics (Doorn, 2019; Schmidt, 2023). For example, researchers on public perceptions of potable water reuse often focus on delineating factors that impact acceptance of such systems (e.g., Dolnicar et al., 2011; Hartley, 2006; Po et al., 2003), implicitly suggesting that values such as safety and health are critical and must be addressed accordingly. However, it remains unclear whether current research adequately addresses these values that are relevant to society. There is reason for concern because the broader water management context has repeatedly been criticised for insufficiently considering the values of society (Correljé & Broekhans, 2015; Harrington et al., 2023; Ravesteijn & Kroesen, 2015). Although an important step towards incorporating values into technological design, these proposed values pertain to various water management practices, including flood management, water provision, and wastewater treatment. Some values may be relevant for one practice but less so for another. Moreover, these values emerged from the societal discourse, whereas the values considered relevant in current research may be significantly different.

As such, we do not yet sufficiently understand which values are deemed pertinent by potable water reuse scholars, how these values are conceptualised, and how they relate to each other. This neglect limits our ability to assess whether potable water reuse systems are designed in a value-sensitive manner, as it remains unclear whether academic values pertinent to potable water reuse align with society’s values. As a result, engineers may unintentionally disregard values, even if they are essential to the social and ethical desirability of potable water reuse systems.

This paper aims to enhance our understanding of potable water reuse values by conducting a large-scale systematic literature review of scientific literature. We employ probabilistic topic modelling to identify latent values and their temporal development. Additionally, we use thematic analysis to interpret values relevant to potable water reuse, providing a value-sensitive perspective on research efforts. This approach enriches the ongoing debate on sustainable water management and encourages responsible development of these systems.

Following this introduction, Sect. “Theory” introduces key concepts like values, VSD, and potable water reuse. We then outline the research methodology, detailing data collection and analysis in Sect. “Methodology”. Section “Results” presents the research findings, while Sect. “Discussion” discusses their implications, limitations, and future outlook. Finally, Sect. “Conclusion” offers concluding remarks.

## Theory

This section outlines the key concepts of this research. Section “Responsible Research and Innovation ” covers Responsible Research & Innovation through Value-Sensitive Design, followed by an in-depth conceptualisation of values (Sect. “Values, value conflicts and value change”). Then, Sect. “Potable water reuse and values” explains the workings of potable water reuse systems and their relationship with values.

### Responsible Research and Innovation

Responsible Research and Innovation (RRI), occasionally referred to as Responsible Innovation, is an umbrella term for inclusive and risk-mitigating approaches to innovation (Burget et al., 2017; Wiarda et al., 2021) that embody one or more elements of the four dimensions of anticipation, inclusion, reflexivity, and responsiveness (Stilgoe et al., 2013). RRI involves a ‘transparent, interactive process’ where stakeholders and innovators work together to ensure the acceptability, sustainability, and societal desirability of the innovation process and its products (Von Schomberg, 2011). The approaches are, thus, based on the idea that including a wide range of different perspectives enhances the quality and ethical acceptability of decision-making and, consequently, allows for a better accommodation of society’s values (Stirling, 2008). RRI seeks to proactively address unexpected and undesirable consequences of innovation through pre-emptive measures (Von Schomberg, 2011). For instance, Taebi et al. (2014) suggest that innovating responsibly should entail the assessment of technology’s ethical and social desirability early on by identifying and embedding values. As such, RRI recognises that innovations are value-laden and that they impose a “vision of (or prediction about) the world” (Akrich, 1992, p. 208). While there are numerous ways in which RRI can be conceptualised and articulated (Fisher et al., 2024), we are specifically interested in the values that are inscribed into the design of potable water reuse systems. It is precisely the approach of VSD that is concerned with identifying and designing for such values (Heezen et al., 2023).

The Value-Sensitive Design (VSD) approach is often considered a cognate and/or a substantiation of RRI (Fisher et al., 2024; Jenkins et al., 2020; Simon, 2017). Both approaches suggest that technology is not simply a material, value-neutral artefact but argue that these technologies reflect the values of designers and engineers and do not necessarily accommodate users’ and society’s needs (van den Hoven et al., 2015a). VSD was developed initially by Friedman, describing it as “a theoretically grounded approach to the design of technology that accounts for human values in a principled and comprehensive manner throughout the design process” (Friedman et al., 2013, p. 56). It rests on the premise that we can uncover relevant values before technology diffuses in society (van de Poel, 2021). Over time, this approach has evolved into sub-forms like Design for Values, proactively integrating values into technology design (e.g. van den Hoven et al., 2015b), and Values in Design, analysing how values are embedded in existing technologies (e.g. Knobel & Bowker, 2011).

### Values, Value Conflicts and Value Change

VSD critically hinges on the notion of values while recognising that they are inherently contested, plural, and open to interpretation. The way values are conceptualised can, therefore, differ substantially across disciplines and studies. For instance, some scholars have conceptualized ‘trust’ as a value (e.g., Nickel, 2015; Vermaas et al., 2010), whereas others view it as a mediator between values (e.g., Gullberg et al., 2023). Categorizations of values are likewise contested. Schulz et al. (2024), for example, differentiate between governance-related values, fundamental values, and assigned values. Other scholars have similarly categorised different types and domains of values (e.g., Shalsi et al., 2024), such as moral values (Quine, 1979), human values (Schwartz & Bilsky, 1987), organisational values (Bourne & Jenkins, 2013), intrinsic values (Kagan, 1998), amongst many others. Against this background, VSD tends to view values as socially constructed concepts that express what is deemed good or desirable. In this research, we specifically focus on ‘public values’, which we understand as matters that are widely shared and sustained by a community and which act as a foundation for collective decision-making (cf., Bozeman, 2007; Jørgensen & Bozeman, 2007). Such values are considered important in life and are shaped by the beliefs and desires of a contextually situated person (Friedman et al., 2013). Yet, values are not merely personally held preferences but reflect an understanding of what is worth striving for within a particular society (van de Poel, 2009). Hence, values are inter-subjective (Taebi & Kadak, 2010) and serve as “orienting judgment devices” for present and future actions (van de Poel & Kudina, 2022, p. 40).

At the same time, accommodating several values can be challenging as they can be conceptualised differently, oppose each other, or change over time. Values are thus not discrete entities (Demski et al., 2015), and prioritising one value can come at the cost of other values (Popa et al., 2023). Such opposing values are referred to as value conflicts (de Wildt et al., 2019) and can occur when “considered in isolation, they evaluate different options as best” (van de Poel, 2009, p. 977). For example, the value of performance often comes at the expense of the value of affordability. Such value conflicts can be addressed through adapting innovations but generally require value trade-offs, meaning one value is to be prioritised over the other (Künneke et al., 2015). In contrast to such value trade-offs, innovations can also overcome value conflicts. For instance, the storm surge barrier for Dutch deltas could resolve the conflict of environmental impacts (sustainability) and provide safety from flooding – values that were previously thought to be inconsumable (Correljé & Broekhans, 2015).

Values are often considered to be universal, stable entities. Yet, they are not necessarily static but are “dynamic, holistic and systemic entities” that are subject to change with societal and technical development (Correljé et al., 2022; van de Poel, 2021). The dynamic nature of such value prioritisation is often referred to as value change, alternatively known as technomoral change (Swierstra, 2013) or moral revolution (Appiah, 2011; Baker, 2019). Scholars studying value change seek to understand how and why values change over time, often with a particular focus on the role of technology development. For instance, Swierstra (2013) suggests that value change stems from moral uncertainty introduced by scientific and technological advancements.

Van de Poel (2021) further suggests that value change can manifest in different forms, such as the emergence of new values, shifts in value prioritisation within a technological design or alterations in value conceptualisations. “[Values] carry over from earlier experiences” (van de Poel & Kudina, 2022, p. 1), and from an evolutionary perspective, one may argue that processes of variation, selection, and retention create emergent value trajectories that are path-dependent but prone to contingency (Wiarda et al., 2024). Value change can be radical (i.e., short-term value change) and incremental (i.e., long-term value change) (Abramson & Inglehart, 2009). Here, short-term radical changes are usually a response to substantial alterations in one’s environment, while decades-long incremental changes are much harder to explain (Manfredo et al., 2017). Changing values can lead to challenges once embedded in designs and society, exemplified by the current difficulties of accommodating sustainability in our transportation systems, a historically overlooked value (van de Poel, 2021). Value change can become problematic for large infrastructures like transportation and water systems, as these systems have been utilised for decades, risking becoming unacceptable if they no longer align with society’s value prioritisation (de Wildt et al., 2022).

### Potable Water Reuse and Values

In this study, we view potable water reuse systems as socio-technical systems that shape and are shaped by their social context. Potable water reuse refers to treating wastewater (previously used by households, industry, or agriculture) and repurposing it (Kayhanian & Tchobanoglous, 2016). Wastewater treatment levels vary based on whether the intended reuse is potable or non-potable. Traditional drinking water systems typically rely on groundwater or surface water, while potable reuse offers an alternative source by treating and recycling wastewater.

Two commonly discussed reuse systems for potable water are indirect potable water reuse (IPR) and direct potable water reuse (DPR)^1^. Both systems consist of complex treatment trains that can be constructed with various treatment technologies, depending on contextual requirements and constraints. With IPR, treated water is discharged into an environmental buffer such as a body of water (e.g., surface or groundwater), allowing for water storage in times of excess and providing additional time to identify contamination incidents (Gerrity et al., 2013). DPR does not use an environmental buffer between the wastewater treatment and the drinking water treatment plant, making it a viable option in regions where buffers are unavailable or inefficient due to high run-off or evaporation rates (Moya-Fernández et al., 2021). The growing similarity in technologies used for IPR and DPR has led to a shift towards a unified concept of potable water reuse (see, e.g. National Research Council, 2012).

Potable water reuse scholars often only address values implicitly, together with other factors of public acceptance, rather than discussing them as standalone entities (see, e.g. Po et al., 2003; Scruggs et al., 2020). Yet, the so-called ‘Yuck factor’ and perceptions of risk (Duong & Saphores, 2015; Leong & Lebel, 2020), knowledge about technologies (Boyer et al., 2017; Khan & Anderson, 2018), and the urgency of addressing water shortages (Scruggs & Thomson, 2017) have been shown to impact public acceptance significantly and indicate that the values of health and safety are deemed essential by society.

Despite the recognised importance of values in water management, a specific value landscape^2^ for potable water reuse has yet to be established. While the broader field of water management increasingly acknowledges the critical role of society’s values (Gullberg et al., 2023; Schulz et al., 2024), historically, designing for values has often been absent or only implicitly considered (Correljé & Broekhans, 2015; Ravesteijn & Kroesen, 2015). The UN’s 2021 ‘Valuing Water’ report criticises this historical neglect and highlights significant underdevelopment in capturing societal and environmental values in water management (Sandhu et al., 2023; Shalsi et al., 2024; United Nations, 2021).

Some prominent studies on values in water management have explored what values are important in the water domain. As discussed, Schulz (2017) works with fundamental values (core personal convictions like power, security, and benevolence), governance-related values (guiding decision-making processes, such as efficiency, sustainability, and solidarity), and assigned values (context-specific values attributed to water resources, like biodiversity, aesthetics, or economic value). Ravesteijn and Kroesen (2015) focus on engineered solutions and differentiate between technology-dependent, management-dependent, and historical cultural-dependent values, highlighting the values of safety, security, sustainability, justice, and participation. Specific technological contexts like reusing coal seam water show a strong focus on technology-dependent values, thereby neglecting societal implications such as justice, conservation and sustainability (Shalsi et al., 2024). Research often overlooks values relevant to society, including cultural understandings of water, justice, and equity concerns (Harrington et al., 2023; Meehan et al., 2020). More recently, justice considerations have gained attention in water ethics (e.g., Doorn, 2019; Schmidt, 2023) and within the Sustainable Development Goals framework (see, e.g., Houngbo, 2023; United Nations, 2022), indicating a potential shift towards broader value considerations.

The inherent complexity of water management in the diverse context of challenges, ranging from scarcity to pollution, makes it difficult to generalise the relevance of specific values. Certain values may be highly relevant to some challenges but less to others, making it difficult to generalise their relative importance. Precisely in such contexts, VSD highlights the need for a well-defined, context-specific value landscape for technological success (Friedman et al., 2013; van den Hoven et al., 2015a). Yet, it is crucial to acknowledge that the values pertinent to a technological intervention like potable water reuse represent a subset of the broader water management value landscape. Our study represents a first step in understanding which public values are reflected in potable water reuse research. This study systematically identifies and conceptualises the values featured in the academic literature on potable water reuse, offering a starting point for understanding which public values are reflected and providing a basis for value-sensitive design.

## Methodology

**Fig. 1:**
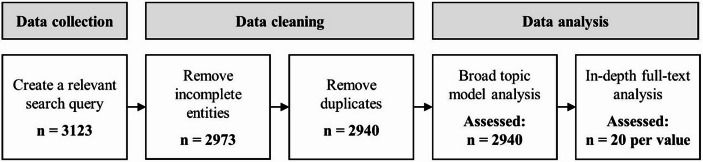
Systematic literature review process

To identify dominant values in potable water reuse research and track how these values have evolved, we conducted a systematic literature review in combination with probabilistic topic modelling. Figure 1 provides an overview of the review process. Section 3.1 covers our data collection, followed by Sect. “Data analysis”, which explains the data analysis.

### Data Collection and Cleaning

We first built a dataset suitable for topic modelling to identify and describe latent values inscribed in potable water reuse research. To do so, we followed the topic modelling guidelines as presented by (de Wildt et al., 2018): (1) choosing an appropriate text corpus, (2) ensuring the sufficient size of the dataset, and (3) restricting the data to the desired research topic.

(1) To assess the current value landscape of academic research on potable water reuse, we used Scopus as our database because it predominantly contains scholarly literature and is the most extensive publication database available (Falagas et al., 2008; Visser et al., 2021).

(2) To make this dataset suitable for topic modelling, the size of the dataset needed to be sufficiently large. A minimum of 1000 entities is often required (van de Poel et al., 2022).

(3) Probabilistic topic modelling research involves text-mining the semantic structures of each document. The dataset must be valid, as no exclusion criteria will apply beyond general data cleaning (e.g., removing incomplete and duplicate entries). Crafting the appropriate search query is critical and requires an iterative process. The desired dataset should be broad enough to capture various studies on potable water reuse but narrow enough to exclude other treatment systems like desalination. Thus, we used terms like “water reuse” and its synonyms, along with “direct potable water reuse” or “indirect potable water reuse” combined with “potable,” “drinkable,” or “drinking.” The used query is as follows:

TITLE-ABS-KEY ((“Water Reuse” OR “Water Recycling” OR “Recycled Water” OR “Direct Potable water reuse” OR “Indirect Potable water reuse” OR “Advanced Water Purification”) AND (“potable” OR “drinkable” OR “drinking”) AND NOT (“Desal*”) AND PUBYEAR > 1989 AND PUBYEAR < 2024).

Our data collection resulted in a dataset of 3123 records, including metadata such as titles, publication years, abstracts, DOIs, and more. Then, we manually removed incomplete entities (without an abstract or author information) and duplicates, leaving 2940 records for our topic modelling^3^.

**Fig. 2:**
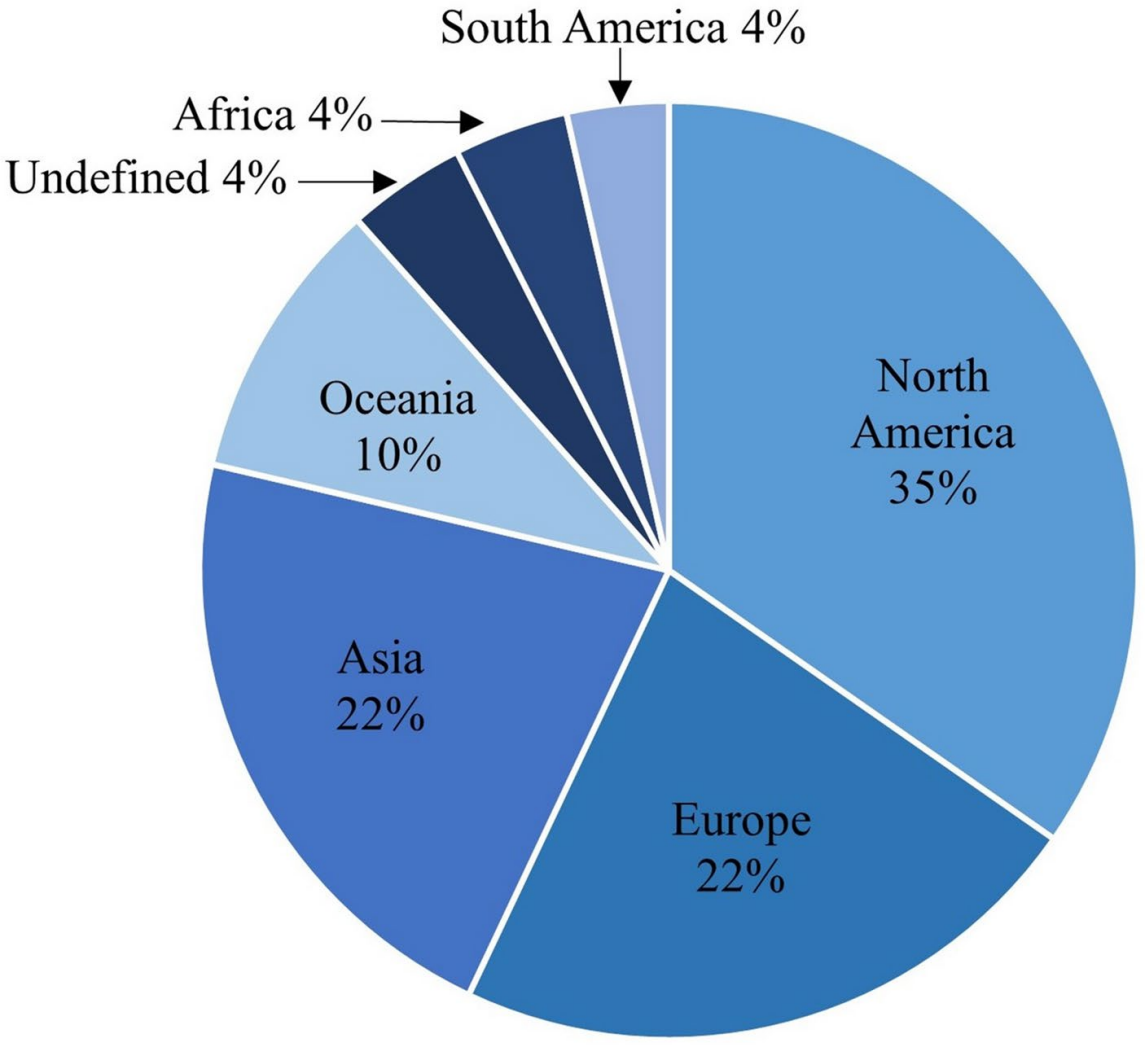
Geomapping the continents of potable reuse institutes

We find that a significant number of our sample’s records is attributed to North America (35%), Europe (22%) and Asia (22%)^4^ (See Fig. 2; Table 1). A considerable share of North American contributions come from the United States (31.3%). Australia is the second largest contributor to our sample, following with a share of 9.6% records; other large contributors come from Asia, such as China (6.7%) and India (3%). Based on our geomapping, one could argue that Western-oriented values may be more represented in the sample (e.g., North America, Europe, and Australia).

**Table 1:** Affiliated countries of institutes researching potable water reuse

Country	[%]
United Kingdom (Europe)	3.5%
Germany (Europe)	3.2%
India (Asia)	3.0%
United Kingdom (Europe)	3.5%
Germany (Europe)	3.2%
India (Asia)	3.0%
Brazil (South America)	2.7%
Canada (North America)	2.6%
Netherlands (Europe)	2.4%
**Rest**	35.1%

### Data Analysis

Using our cleaned dataset, we conducted topic modelling to identify dominant values in potable water reuse systems (Sect. “Identifying values”) and conceptualised these values using thematic analysis (Sect. “Conceptualising values”).

#### Identifying Values

Values are often latently discussed in texts (de Wildt et al., 2018). For instance, when referring to the value of justice, authors do not necessarily use the term justice. Instead, they may use synonyms, cognates, or closely related words that collectively refer to the value of justice (e.g., fairness, equity, equality, etc.). Hence, analyses that solely focus on the word justice may omit essential records that are relevant to our understanding of the value, rendering value identification through keyword-based searches difficult. Using multiple closely related keywords can help identify latent values but commonly results in a higher number of irrelevant documents (de Wildt et al., 2022). For example, the word ‘just’ could also refer to a phenomenon that ‘something just happened’.

To address this challenge, we used a software package called ValueMonitor^5^, developed to identify latent values from records, and which relies on a widely used probabilistic topic modelling software called Corex (Gallagher et al., 2017). Topic modelling is a form of text-mining that considers the distribution of words when identifying latent concepts. Each word is assigned a certain weight, corresponding with the likelihood that specific values are found in records. Continuing with the same example, when the word justice is given a relatively low weight, associated words such as equity and fairness will need to receive a high weight before the software concludes that a record indeed refers to the value of justice.

The creation of word distributions is an iterative process for which keywords are fed into the software to guide specific topic formulations (de Wildt et al., 2022). The ValueMonitor was developed to identify public values across various domains, of which the more detailed theoretical underpinnings have been extensively described and tested in other studies (cf., De Wildt et al., 2022; van de Poel et al., 2022; Wiarda et al., 2024). The tool was developed for the European Research Center to investigate the latent values embedded within emerging technologies.

ValueMonitor’s language model currently contains word distributions for 28 different public values (see Appendix I for definitions). Generally speaking, topic modelling works more reliably when using texts of roughly 50–300 words to identify these latent values and trace their prevalence over time, as van de Poel et al. (2022) demonstrated. We utilized ValueMonitor to identify latent values from our records’ abstracts. After identifying values, we use the publication dates of each record to trace the values over time. This yields insight into the relative importance of different values over the last 30 years.

#### Conceptualising Values

After the ValueMonitor identifies prevalent values in the dataset, we manually review the full text of the top 20 most cited articles for each value, as these are likely the most influential in constructing the contextual conceptualisation in the field. We applied an inductive thematic analysis using open and axial coding and categories, where we first searched for the respective values within the text and then identified relationships and patterns among them. For open coding on the sentence level, we utilise value definitions provided by ValueMonitor as our coding rules. For instance, the value of justice is described as “fair and equal treatment”. Here, one author conducts the thematic analysis for each value, after which the remaining authors check the themes against the coding rules for validity. We also examine whether these articles link values to capture potential relationships. While this step is not meant to be an exhaustive analysis of value relationships, we use it exploratively to understand better how values are entangled in research.

## Results

Section “Identifying and tracing pertinent values” presents the most frequently mentioned values in potable water reuse literature and their changing prevalence. Section “Understanding pertinent values” explores the dominant conceptualisations, whereas Sect. “Value conflicts and complementarities” examines value conflicts and complementarities.

### Identifying and Tracing Pertinent Values

**Fig. 3:**
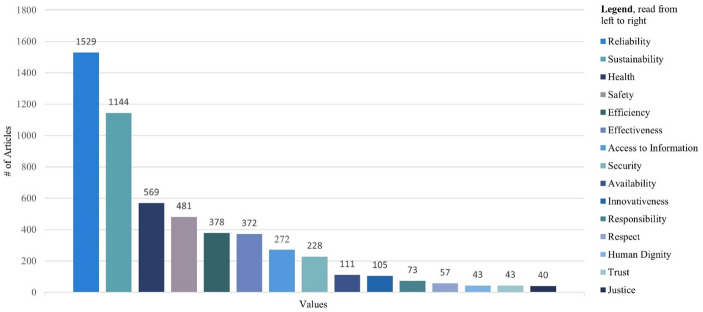
Values and their respective frequency for potable water reuse research

As seen in Fig. 3, reliability and sustainability are the most frequently occurring values in this data set, with 1529 and 1144 document counts, respectively. Moreover, health, with 569 and safety, with 481 counts, seem to be highly common values for water reuse systems. As these four values seem most influential in this case, we will examine how they are conceptualised in the literature.

**Fig. 4:**
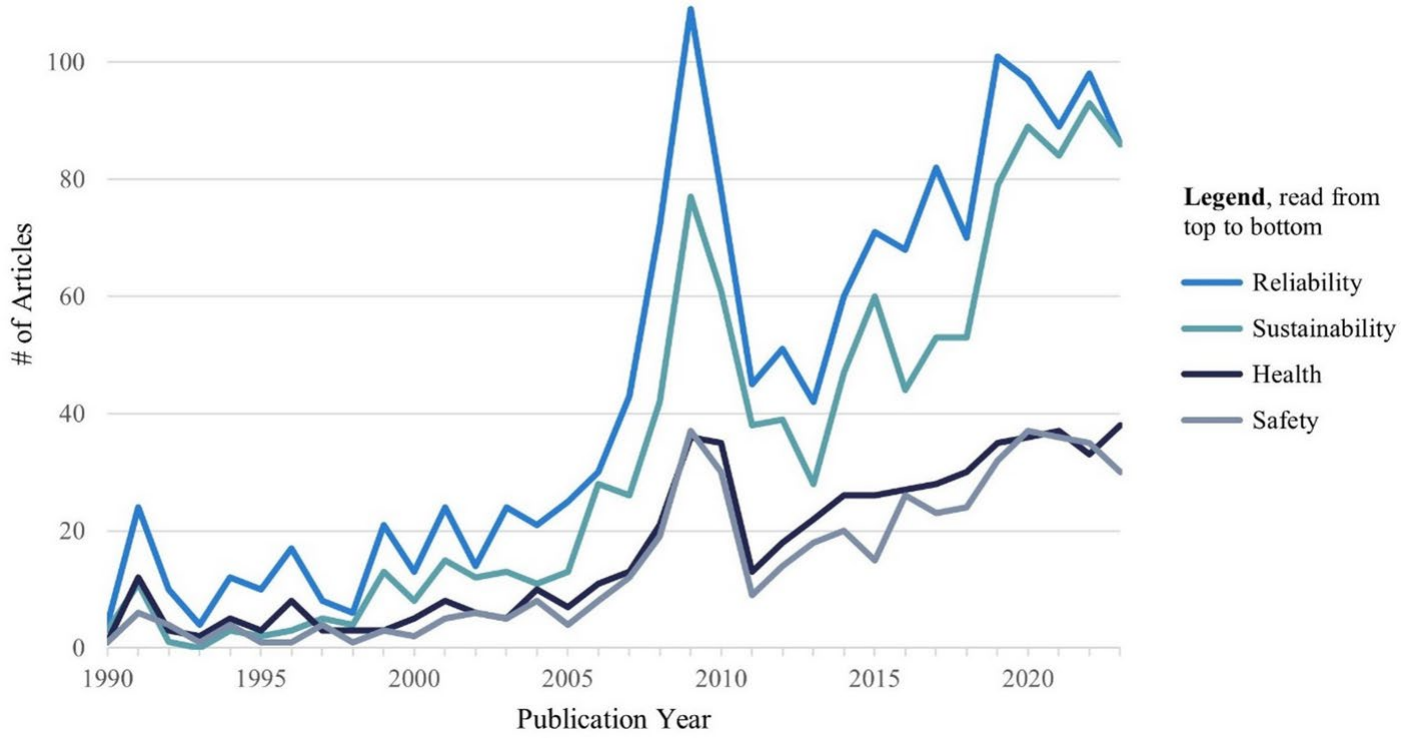
Value dynamic of potable water reuse between 1990 and 2023

If we trace the prevalent values over time (see Fig. 4), we observe a stark growth in all values proportionally corresponding to the increase of potable water reuse studies. The results suggest that the relative importance of the four key values has remained roughly consistent over time, with reliability and sustainability being significantly more prominent than health and safety. As such, societal debates and changing requirements do not seem to have affected these values over time. The precise cause of this peak is unclear, although it may speculatively be linked to increased global discussions around the human right to water, with the release of the IPCC’s Fourth Assessment Report in 2006/2007 (IPCC, 2007), and a UN Special Rapporteur appointed in 2008 (Human Rights Council, 2008), which could have stimulated research on water-related challenges.

### Understanding Pertinent Values

#### Reliability

Population growth and the exacerbating effects of climate change, such as more extreme and longer-lasting droughts, “make traditional supplies unreliable” as a source of drinking water (Warsinger et al., 2018, p. 211). In this context, reliability is measured by a region’s climate and the ability of a specific water source to meet water demand.

Yet, the ValueMonitor reveals that the concept is predominantly linked to another conceptualisation: the water reuse system’s performance. For example, Tang et al. (2018) postulate that water reuse “[…] offers a reliable and sustainable solution to cities and regions facing a shortage of water supply” (p. 10215). In such a context, the system’s reliability is often directly associated with the ability of a system to produce high-quality water, meaning that drinking water quality standards are sufficiently met (see Ahmad et al., 2003; Tang et al., 2018). However, these standards are criticised as outdated and needing revision, especially regarding emerging organic contaminants (Pal et al., 2014).

Reliability is also a recurring concept in membrane research, particularly in addressing the common issue of fouling. Membranes are pretreatments within wastewater treatment processes and are regarded as potentially viable solutions, receiving much attention (e.g. Bellona et al., 2004; Lutchmiah et al., 2014; Warsinger et al., 2018). Filtration membranes are often threatened by fouling. Consequently, scholars have researched various pretreatment options and membrane materials to decrease the fouling rate (e.g. Ahmad et al., 2003; Kimura et al., 2004). Yet, membrane filtering is generally “less reliable for water treatment than physical sieving” for contaminant particles smaller than the membrane pores (Huang et al., 2009, p. 3012). Moreover, inconsistent study results on the reliability of contaminant removal, incomplete tracking of substances, and the uncertainty about the effect of emerging contaminants shed doubt on the performance of particular water treatment trains (Escher et al., 2014; Le-Minh et al., 2010).

#### Sustainability

The value of sustainability has several meanings in the context of water reuse systems but specifically refers to environmental sustainability. Drinking water is becoming increasingly scarce (Gomes et al., 2017; Oki & Kanae, 2006) because of climate change and water pollution with heavy metals, biological agents, and other miscellaneous substances (Gupta et al., 2009). This trend motivates research to understand better how to remove pollutants without further harming the environment through byproducts. As Li et al. (2008) put it: “The challenge to achieve appropriate disinfection without forming harmful disinfection byproducts by conventional chemical disinfectants […] calls for new technologies for efficient disinfection and microbial control” (Li et al., 2008, p. 1). Various absorbents have been proposed to combat pollutants (Gupta et al., 2009), such as bioreactor technologies (Melin et al., 2006; Yang et al., 2017), forward/reverse-osmosis membranes (Lutchmiah et al., 2014; Tang et al., 2018) and biochar (Inyang & Dickenson, 2015).

Water reuse systems are “critically examined according to specified criteria for performance and sustainability” (e.g., quality of treatment, energy usage; Sobsey et al., 2008, p. 4261), facing at least three types of sustainability challenges. First, sustainability may be affected by a lack of the value ‘effectiveness’. Authors report cases in which water treatment technologies inadequately remove artificial sweeteners (Scheurer et al., 2009) and antibiotics, the latter leading to bacterial resistance and the contamination of local surface, ground, and drinking water (Fick et al., 2009; Le-Minh et al., 2010). Second, the treatment technologies themselves may also be harmful. While carbon nanoparticles can remove bacterial pathogens, natural organic matter, and cyanobacterial toxins (Li et al., 2008), particular water filters can severely affect aquatic life and our food chain when released into the environment (Upadhyayula et al., 2009). Third, water reuse systems can create unsustainable byproducts such as residual sludge; how this waste can be managed sustainably remains unclear. However, Babatunde and Zhao (2007) propose that residual sludge should be used for several purposes, including construction materials and land-based applications in the future.

#### Health

The concept of health can be related to two distinct subjects, humans and the environment, where the former has received significant attention. Moe and Rheingans (2006) suggest that human health is related to water for drinking, hygiene and feed production. Water affects public or human health, but “poor and disadvantaged populations are the ones who will suffer most from the negative effects of climate change on water supply” (DeNicola et al., 2015, p. 1).

In water reuse systems literature, a significant fraction assesses the challenges related to physical health. Bruce et al. (2010) draw attention to the “public health significance of trace levels of pharmaceuticals in potable water […] particularly with regard to the effects of long-term, low-dose exposures” (p. 5619). For example, Xi et al. (2009) express concerns over current water treatment systems that cannot effectively remove antibiotic-resistant bacteria and argue that these bacteria could subsequently spread via potable water distribution systems. Other scholars are concerned with disinfectant byproducts and their potential health impacts (Krasner, 2009). Moreover, uncertainty about the health implications of trace organic contaminants such as pharmaceuticals and personal care products remains due to the lack of long-term research data (Alexander et al., 2012). As a result, wastewater treatment methods, such as coagulation-based processes or plasma-based water purification, are tested on their adequateness in removing such contaminants (e.g. Alexander et al., 2012; Foster, 2017).

Next to physical health, some scholars suggest that health concerns are linked to social acceptance and environmental sustainability. For example, in the United States, public debates reflect unease about the health implications of using water reuse systems (Hartley, 2006). Hartley (2006) observes that even within the scientific and technical communities, these debates are not simply resolved as they “disagree over the public health viability of indirect potable water reuse, with major water resource professional associations and respected research and expert panels taking opposing positions” (p.117). At the same time, the potential impacts of contaminants are prone to high degrees of uncertainty (Reungoat et al., 2010). Studies have, therefore, examined the environmental effects of pollutants produced or not removed by wastewater treatment methods (see, e.g. DeNicola et al., 2015; Pal et al., 2014).

#### Safety

The goal of any drinking water treatment plant, irrespective of whether it is part of a water reuse system, is to provide safe drinking water. In this context, safety appeals to the composition of water – devoid of pollutants. Research primarily aims to understand the long-term impacts of pollutant exposure better, but this appears challenging due to limited data availability (see, e.g. Bruce et al., 2010; Rayne & Forest, 2009).

The value of safety is applied to either understanding particular contaminants (see, e.g. Cacciò et al., 2003; Johnson et al., 2008; Rayne & Forest, 2009) or assessing and developing methods to remove these (see, e.g. Fanourakis et al., 2020; Upadhyayula et al., 2009; Westrick et al., 2010). For example, Cacciò et al. (2003) found a presence of giardia cysts in several Italian wastewater systems after treatment, which “increase the risk of human infection with these pathogens” (p. 3397). From a methodological perspective, Upadhyayula et al. (2009) criticise the use of carbon nanotube technologies for their potential cytotoxicity, which induces “drastic safety and environmental impacts” (p.10). Moreover, impact assessments of other cytotoxic contaminants (e.g., chemotherapeutic drugs) on the aquatic environment are severely lacking (Johnson et al., 2008). Scholars, therefore, advocate for regulations that help safeguard the environment and urge to “[…] first consider the safety of receiving water bodies” (p.550) before turning to water reuse systems (Qu & Fan, 2010, p. 550).

The value of safety is also discussed in relation to public perceptions, acceptance and system security. For example, de França Doria (2010) claims that factors such as trust in authority, familiarity with tap water, and perceived water quality contribute to the public’s perceived risk. Research shows that risk perception is highly influenced by trust in executing authorities (Ross et al., 2014). Moreover, safety perceptions seem to be intricately linked with social acceptance. Supporting this, a study on online shopping behaviour found that consumers’ acceptance of recycled water products relies heavily on safety perceptions provided by consumers’ reviews (Fu et al., 2020).

### Value Conflicts and Complementarities

With the key values identified and conceptualised, we can examine their relationships – value conflicts and value complementarities^6^. Value conflicts often arise when prioritizing one value results in compromising another. Our research identified a significant value conflict within the study of specific technologies used in potable water reuse systems, illustrated by a critical and extensively researched treatment stage: membrane filtration.

Membranes are increasingly vital elements in waste and drinking water treatment systems. Although they are considered to produce low-cost (affordability), high-quality water (safety) with a low carbon footprint (sustainability), membrane fouling remains a significant challenge (Huang et al., 2009). This accumulation of contaminants on membrane surfaces can jeopardise the long-term quality of the produced water (reliability) (Tang et al., 2018). To overcome this challenge, extensive research has focused on optimizing the membrane design and adding another treatment step, introducing complexity to the treatment system. For example, membrane pretreatments have been shown to provide superior reliability but often necessitate additional non-reusable chemicals (Huang et al., 2009), are highly energy intensive (Tang et al., 2018), or create toxic sludge (Babatunde & Zhao, 2007), again impacting the treatment system’s sustainability.

These examples show that optimizing for one value must be balanced with other relevant values in water treatment technology. The currently proposed technologies and approaches prioritize reliability over sustainability, making them appear incompatible. However, ongoing research aims to overcome this dichotomy, indicating rising awareness about the importance of sustainability. For example, advancements in membrane technology employing biodegradable materials or innovative pretreatment processes reducing chemical usage and energy consumption are being explored to balance these competing values (see e.g., Li et al., 2008; Lutchmiah et al., 2014).

Moreover, this research shows that several values are intricately interlinked, where the promotion of one value triggers a chain reaction affecting others. We refer to these relationships as value complementarities which particularly arose when considering the challenge of effectively removing contaminants, thereby explicitly affecting the safety of the produced drinking water. The complementarity between reliability and safety is widely recognised, though often implicitly addressed through the enhancement of specific technologies (e.g., Kimura et al., 2004; Warsinger et al., 2018). Furthermore, safety concerns can also extend to sustainability concerns of these systems, particularly regarding the environmental impact of discharging inadequately treated wastewater. For instance, releasing contaminants such as carbon nanotube particles (Upadhyayula et al., 2009) or cytotoxic substances used in chemotherapy (Johnson et al., 2008) can cause significant harm to the aquatic environment. Lastly, the inadequate removal of contaminants not only impacts sustainability but can also pose health concerns. For example, contaminants can enter the human body through drinking, promoting the development of antibiotic resistance in humans (Fick et al., 2009; Le-Minh et al., 2010) or accumulate in the food chain, inducing additional health risks (Upadhyayula et al., 2009).

Thus, value complementarities are often observed when one particular value is harmed, leading to a chain reaction that affects other values. Safety emerges as a central concern in potable water reuse systems, playing a critical role in supporting both health and sustainability, while reliability is vital to maintaining safety.

## Discussion

This paper offers a value-sensitive perspective on water reuse by identifying and conceptualizing pertinent values in research. We identified four most pertinent values in research on potable water reuse, namely:

• *Reliability*: interpreted at the system level as increased water availability but is mainly used in the context of the ability of water reuse systems to produce ‘high-quality’ drinking water standards by effectively removing contaminants.

• *Sustainability*: understood as the environmental impact of technology. It appears critical for overall water availability but is mainly associated with effectively removing pollutants before releasing treated wastewater into the environment.

• *Health*: relates to human and environmental well-being, focusing on the threats posed by pollutants. Research often emphasises treatment systems that eliminate harmful contaminants, as health concerns drive public concerns about water reuse.

• *Safety*: addresses specific pollutants remaining in treated water. Most research aims to identify and remove these contaminants, while some emphasise the relationship between safety and public perception.

Research primarily focuses on the ability of potable water reuse systems to produce safe, high-quality water, which is critical for health and sustainability but remains challenging. Notably, we see that the pertinent values are often conceptualised in a technical manner. This observation is unsurprising, as this review revealed that most academic research on potable water reuse focuses on developing and optimizing treatment technologies, which aligns with findings from previous studies that examine values in the broader field of water management (see Ravesteijn & Kroesen, 2015; Shalsi et al., 2024). A reason for this technocentric focus may be that choosing technological interventions, like potable reuse, often reflects an underlying anthropocentric worldview from a monodisciplinary perspective that implicitly prioritises values associated with human control and manipulation of natural systems (see Schulz et al., 2017).

**Fig. 5:**
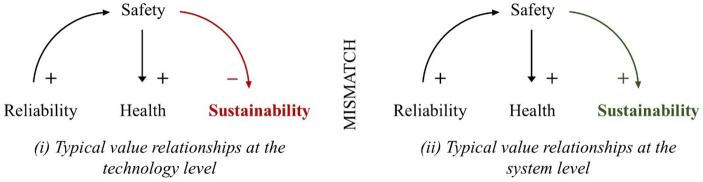
Schematic of typical value relationships. [+ denotes value complementarities, and – denotes value conflicts]

We furthermore analysed the relationships between the four most recurring values in academic research. Reliability, safety, and health are widely recognised as complementary, while the relationship between safety and sustainability is somewhat ambivalent, at times conflicting and at others complementary. The nature of the relationship between safety and sustainability appears to depend on the research perspective (illustrated in Fig. 5). Here, especially the value of sustainability appears to be defined in various ways, which does not necessarily imply direct value conflicts but could also be symptomatic of different understandings of the concept. As such, concept clarity is essential to mitigate ambiguity.

Technology-level research focuses on developing or optimizing specific technologies used in potable water reuse systems. From this perspective, engineers seem able to design for reliability, safety, and health effectively, where improving one value benefits the others. However, broader sustainability issues may be overlooked. System-level research emphasises the broader implications of these values, such as overall environmental and human impacts, but can overlook technical limitations. Given the dominance of technology-level research, these broader sustainability aspects are likely underemphasised and raise the question of to what extend academic values reflect societal concerns.

### Does Academic Research Align with Society-Level Values?

At first glance, the prevalence of reliability, sustainability, health, and safety suggests a reasonable alignment of research with societal concerns. However, this apparent alignment masks discrepancies and complexities in conceptualising these values. While research addresses these values, it does so primarily through a technical lens. Although this is important for developing effective treatment systems, other critical social aspects may be overlooked – a concern supported by broader critiques of technology development (van den Hoven et al., 2015a; Winner, 2017). For example, studies on public acceptance and water security emphasise the perceptual and social dimensions of health and safety (see., e.g., Duong & Saphores, 2015; Harrington et al., 2023; Meehan et al., 2020). Instead, academic literature on potable reuse primarily frames these values in terms of technical performance and contaminant removal. Although acknowledged by some scholars (see e.g., de França Doria, 2010; Ross et al., 2014), the focus on technological optimization overshadows some of these society-level concerns. This suggests a disconnect where some values in research and society seem to align, but their overall conceptualisation can differ considerably. Therefore, one of the implications of our work is that actors should work simultaneously with technology-level and system-level values (conflicts) but also recognise and respond to value considerations raised by society.

Furthermore, the geographical representation of potable water reuse research hints that predominantly Western values are embedded in research since values and their conceptualisation are culturally and contextually bound. More broadly, existing studies on public perception and acceptance are also primarily conducted in Western regions (see, e.g., Distler et al., 2020; Khan & Anderson, 2018; Ormerod, 2019; Santos et al., 2022). We argue that current research is more likely to reflect Western values. Potable reuse systems are also applied in non-Western contexts such as Africa and Asia. Therefore, future research (see also Sect. 5.3) should actively incorporate diverse perspectives and non-Western values for a more equitable understanding of the potable reuse value landscape in those contexts.

Our research hints at the fragmented nature of the value landscape of potable water reuse. Although our comparison is not exhaustive, it illustrates possible neglected values and different understandings of research and society-level values. It marks a step toward understanding the values considered in current research versus those held by the affected publics. For example, the potential mismatch between technology-level, system-level and society-level values can lead to conflicting recommendations, which highlights the impact that different disciplinary perspectives and value interpretations can have on technology development. Policymakers influenced by technology-centric studies may favour immediate technical fixes, while those guided by system-level research might push for under-researched sustainable advancements. Socially-driven research, on the other hand, may advocate for more research into social aspects of safety. Addressing these mismatches requires transdisciplinary approaches to align research priorities across communities. Moreover, the context-dependent nature of public values necessitates value-sensitive approaches on a case-by-case basis. Van de Poel’s (2013) VSD-informed Values Hierarchy can be the first step in bridging different values and conceptualisations and translating them into concrete design requirements.

### Limitations and Future Outlook

For the use and interpretation of our work, it is important to point out some potential limitations. First, while topic modelling and thematic analysis have been used in other studies to aid the identification and conceptualisation of latent values, it remains important to stress that such results are value-laden constructs themselves. Epistemological limitations in empirical ethics generally stem from implicit interpretation and confirmation biases, as pre-existing conceptions may influence results. This research is likely no exception, but we have tried to mitigate biases by building upon a set of public values that have been used in various VSD studies (see, e.g., de Wildt et al., 2022; van de Poel et al., 2022; Wiarda et al., 2024), and by combining existing systematic, qualitative, and quantitative research methods engaging in reflexive multi-author discussions that aimed to enhance the inter-coder validity of this work. As also discussed in Sect. 2.2., we recognise that any conceptualisation of values is inherently prone to contestation.

Second, although the ValueMonitor tool has been validated in other contexts, it may introduce an ‘anchor bias’ by focusing on a pre-defined set of public values (see Appendix I). While this allowed for a systematic analysis, it might have overlooked values unique to the potable water reuse context. More research is needed to deepen our understanding of values in water management and, specifically, for potable water reuse to enable a more comprehensive analysis.

Third, using the frequency of values as a proxy for their importance has its limitations. This approach allowed us to get a deeper understanding of the four most prevalent values, but other relevant values might be underrepresented in our analysis. Moreover, the frequency of mention may reflect scholarly trends or ease of operationalization, but it does not necessarily reflect the greatest significance in water management. Certain values might be less frequently discussed in academic literature due to their abstractness, their inherent complexity, or current research trends. Yet, they may hold substantial weight in the underlying research goal. Therefore, the four values highlighted in this study should indicate prevalent values in the literature which are not necessarily the most important or comprehensive set of values at play.

Lastly, it is important to acknowledge that our comparison between academic and society’s values is preliminary due to the incompleteness of the full societal value landscape relevant to water management, specifically potable water reuse. While we have drawn upon existing literature to highlight key values in water management practices, especially with regard to engineering solutions, a comprehensive and empirically grounded assessment of societal values in this context is still lacking. Therefore, the identified misalignments should be interpreted as indicative and suggestive, requiring further research.

## Conclusion

Water management research has been criticised for not sufficiently considering values relevant to society in the design of systems. While recent studies made valuable contributions to our understanding of values in specific technologies and water management at large, the value landscape of potable water reuse systems remains underdeveloped. This study investigated the alignment between society’s values and those prioritised in academic research on potable water reuse. Employing a mixed-methods approach, combining large-scale topic modelling with thematic analysis, we identified and analysed latent values within a large corpus of scholarly literature. Our study revealed that academic research predominantly focused on four key values: reliability, sustainability, health, and safety. The thematic analysis discovered diverse conceptualisations of these values. Here, we found that reliability is critical for safety considerations while safety, in turn, is instrumental to ensuring health. Moreover, sustainability showed an ambivalent relationship to the value of safety, which is either conflicting or complementary, depending on the research perspective. While seemingly aligned with public concerns about health and safety, our analysis revealed critical differences. The academic literature primarily understands these values from a technology-level perspective, discussing contaminant removal and system performance. In contrast, studies addressing public perception highlight the importance of non-technical aspects, such as cultural-relativistic understandings of risk, justice, equity, and the cultural significance of water. These society-level values are often overlooked in the predominantly technocentric academic discourse. This misalignment between technology-driven academic research and the broader spectrum of society’s values highlights the need for a more value-sensitive approach to potable water reuse. Although current research contributes to the technical feasibility of these systems, *responsible potable water reuse* requires a broader strategy. Building on our research approach, future research should contribute to a better understanding of the complexity, comprehensiveness and spatial distribution of values in potable water reuse systems to better develop solutions that are technically sound, ethically robust, and socially responsible.

## Appendix I – Value Definitions Used by ValueMonitor

**Table:** 

Value	Definition used by ValueMonitor
*Access to information*	The public’s right to receive environmental information held by public authorities
*Accountability*	Goals and intentions behind relevant decisions can be understood even from an outside perspective and those involved are held responsible for these decisions
*Affordability*	Being cheap enough for people to be able to buy
*Autonomy*	Capacity to act on one’s desires
*Availability*	The accessibility and readiness of resources, services, and opportunities for individuals and communities within a society.
*Beneficence*	Promoting well-being/human flourishing
*Competitiveness*	The ability of a technology to offer an economic advantage.
*Democracy*	Widely and fairly distributed control over decisions
*Effectiveness*	The measure of the degree to which an individual, system, product, or process achieves its intended outcomes or goals.
*Efficiency*	A high effective operation as measured by a comparison of production and cost (as in energy, time, and money).
*Freedom*	Limiting the power that a person or organization has to interfere in your life and to frustrate your desires
*Health*	Contribution to physical and mental well-being.
*Human dignity*	Respect for human life
*Inclusiveness*	The consideration of everyone’s interests
*Innovativeness*	The advancement of new technologies and technological solutions.
*Justice*	Fair and equal treatment
*Privacy*	Being able to control who can see or use information about you
*Profitability*	The ability of a technology to generate financial returns.
*Public participation*	The possibility for direct and indirect stakeholders to share their views freely and be involved in decision-making
*Reliability*	Consistent quality over time
*Respect*	Honouring the dignity, rights, and interests of all stakeholders.
*Responsibility*	(1) The agent needs to be in control of what he/she is doing; and (2) the agent needs to know what he/she is doing
*Safety*	Protection against unintended harm
*Security*	Protection against intended harm
*Solidarity*	The willingness to support members of a group besides the individual directly affected
*Sustainability*	Appropriate access and utilization of biological resources
*Transparency*	Goals and intentions can be understood even from an outside perspective, with those involved at upper levels held clearly accountable
*Trust*	Human confidence in consistent quality over time

For more information, please visit https://valuemonitor.eu/.

## Data Availability

Manuscript data is included as supplementary material.

## Footnotes

1 We deliberately exclude desalination systems from our scope because these systems use different technologies than IPR and DPR, and are often used in different geographical and socio-economic contexts.

2 Following Schulz (2024) the term “value landscape” is used here to denote the pluralistic and often interconnected set of values relevant to a particular domain, acknowledging that these values can be conceptualised and prioritised differently.

3 The dataset is available upon request.

4 This analysis is based on the raw dataset provided by Scopus.

5 https://valuemonitor.eu/.

6 These value conflicts and complementarities are based on the four most frequently identified values. A reflection on the limitations of this methodological decision is given in Sect. “Limitations and future outlook”
